# Knowledge and Practice of Pediatricians Regarding Hypovitaminosis D—A Survey across 33 European Countries

**DOI:** 10.3390/children9121831

**Published:** 2022-11-26

**Authors:** Davor Petrović, Edita Runjić, Ivan Buljan, Antonia Jeličić Kadić, Joško Markić

**Affiliations:** 1 Department of Pediatrics, University Hospital of Split, Spinciceva 1, 21000 Split, Croatia; 2 School of Medicine, University of Split, Soltanska 2, 21000 Split, Croatia

**Keywords:** vitamin D, hypovitaminosis D, knowledge, pediatrics, surveys and questionnaires

## Abstract

Hypovitaminosis D has been recognized as a worldwide pandemic, but there are disagreements regarding its diagnosis and treatment. This study aimed to evaluate the knowledge and practice of European pediatricians concerning vitamin D (VD) and hypovitaminosis D and their adherence to relevant guidelines. A cross-sectional study was conducted through an anonymous survey via SurveyMonkey on 304 European pediatricians. Most of the participants were general pediatricians, followed by endocrinologists and neonatologists. ESPGHAN’s and the national guidelines were the most frequently used. VD testing was mostly performed in patients with skeletal, chronic kidney, and autoimmune diseases. Participants predominantly answered the questions regarding the definition of hypovitaminosis D and VD supplementation correctly, especially in children younger than 12 months. They showed the least knowledge regarding the cut off-point for the initiation of the therapy and therapeutic doses used to treat confirmed hypovitaminosis D. Participants’ conflicting answers could be related to the differences between the guidelines. We consider that experts in this field should create uniform guidelines, and that those guidelines should also be promoted by national or local pediatric associations.

## 1. Introduction

During the previous decade, the scientific community has been increasingly interested in the health benefits of vitamin D (VD) and its role in the treatment of children with kidney, respiratory, neurological, and immunological diseases, as well as in other conditions [1,2,3,4,5,6,7,8,9,10,11,12,13].

Hypovitaminosis D, especially VD deficiency, has recently been recognized as a worldwide epidemic, even a pandemic, affecting the entire population [14,15,16,17]. However, some authors indicate that the reported prevalence of hypovitaminosis D, especially VD deficiency may be overdiagnosed [18,19,20,21,22,23]. Serum concentrations of 25-hydroxyvitamin D3 (25(OH)D) are considered the foremost indicator of overall vitamin D status [1], however an international debate about recommended 25(OH)D levels in serum is still ongoing [24]. Disagreements on the prevalence of hypovitaminosis D in the population are a consequence of disagreements between authors regarding the cut-off value for the diagnosis of hypovitaminosis D. Hypovitaminosis D can be divided into two categories: VD deficiency and VD insufficiency [14]. Different medical societies use different reference values for hypovitaminosis D [19,20,25,26,27]. Nevertheless, a serum 25(OH)D level below 50 nmol/l is generally recognized as hypovitaminosis [20,26,28,29,30,31].

Previous surveys among physicians regarding VD showed uncertainties and gaps in knowledge of physicians about the role of VD, hypovitaminosis D definition, and consequently VD treatment and supplementation [10,32,33,34,35,36].

In the pediatric population, VD is usually administered in two different regiments. For infants and healthy individuals with a risk for hypovitaminosis D, VD is given as prophylaxis, i.e., as prevention of hypovitaminosis D. Risk factors include dark skin, inadequate sun exposure (excessive use of sunscreen with high SPF, staying indoors for much of the day, wearing clothes covering most of the skin, living in northern latitudes), and obesity [27]. That way of administering VD is also referred to in the literature as prophylactic, supplemental, or even maintenance therapy. For the patients with verified hypovitaminosis D, VD is given using different administration regimens and higher therapeutic doses. That way of administering VD is referred to as therapeutic, replacement therapy, or just with the term treatment with VD [19,26,27].

Our aim was to evaluate the knowledge and practice of European pediatricians concerning vitamin D, diagnosis of hypovitaminosis D (VD deficiency and insufficiency), and VD treatment and supplementation. 

## 2. Materials and Methods

### 2.1. Study Design

This study was an online cross-sectional study conducted between November 2018 and February 2019 through an anonymous survey via SurveyMonkey (SurveyMonkey Inc., Palo Alto, CA, USA). The study was conducted according to the Strengthening the Reporting of Observational Studies in Epidemiology (STROBE) statement for reporting cross-sectional studies. The data gathering was conducted online. The study was conducted in accordance with the Declaration of Helsinki, and approved by the Ethics Committee of the University of Split School of Medicine. Approval Code: 003-08/18-03/0001. Approval Date: 29 November 2018.

### 2.2. Survey Sample 

This study included pediatricians who were members of the European Pediatric Association, the Union of National European Pediatric Societies and Associations (EPA-UNEPSA). EPA-UNEPSA consists of 39 pediatric associations from 35 European countries. The invitation email was sent to all associations. 

### 2.3. Participant Recruitment

An identical initial e-mail invitation was sent to the presidents of all European associations. If there was no response, 3 reminders were sent to the presidents, one every week after the initial e-mail, until they positively responded or indicated that they do not wish to assist with the study. The presidents who responded have been asked to either provide us with contacts of pediatricians that are members of their society or to forward the link to the survey on SurveyMonkey to their members. 

### 2.4. Survey Instrument

The questionnaire was based on a questionnaire published by Bonevski et al. [33] and was modified according to the objectives of this study. The original questionnaire was written in the English language, reviewed by 20 Croatian pediatricians, and modified based on their suggestions. The survey has 26 items arranged in four sections. 

The first section of the survey included eleven questions about respondent’s general information and information regarding their education about VD and hypovitaminosis D. The second set of four questions was about VD supplementation, the third section included five questions about the diagnosis of hypovitaminosis D and the final section included 6 questions about hypovitaminosis D treatment. In the questionnaire, we mostly used single select multiple choice questions, with 5 multi-select multiple choice questions (there was an indication prior to that type of question). 

This was an anonymous study, and the IP addresses of participants were not collected to ensure their anonymity. All data were initially collected and stored online via SurveyMonkey, and accessible only to the survey administrator. Before accessing the survey, participants received information about the study and by clicking on the survey link, they provided their consent for participation.

### 2.5. Analysis

Microsoft Excel (Microsoft Corp, Redmond, WA, USA) was used for descriptive analysis. Descriptive statistics were used to describe respondents’ general information (sub-specialization, country of practice, age, and workplace) along with the respondent’s knowledge about vitamin D. 

Participants’ knowledge was assessed based on a share of correct answers to certain questions. Correct answers were defined according to the guidelines by the Institute of Medicine (IOM) [20], Central European Scientific Committee [25], United States Endocrine Society [26], European Society for Pediatric Gastroenterology Hepatology and Nutrition (ESPGHAN) [27], Global Consensus Recommendations on Prevention and Management of Nutritional Rickets [37], Misra et al., guidelines [38]. All answers that did not follow the listed guidelines were considered incorrect. 

To define correct answers regarding the diagnosis of hypovitaminosis D following 25(OH)D levels were used according to the guidelines: 

Central European Scientific Committee and the United States Endocrine Society guidelines [25,26]: VD deficiency is defined when the 25(OH)D level is below 50 nmol/L (20 ng/mL), insufficiency when the 25(OH)D level is between 50 and 75 nmol/L (20–30 ng/mL), and target concentration for optimal 25(OH)D effects at 75–125 nmol/L (30–50 ng/mL) 

Institute of Medicine (IOM), ESPGHAN’s and Consensus Statement on rickets management (Global Consensus Recommendations on Prevention and Management of Nutritional Rickets) guidelines [19,20,27,37]: VD deficiency is defined when the 25(OH)D level is below 30 nmol/L (12 ng/mL), insufficiency when the 25(OH)D level is between 30 and 50 nmol/L (12 and 20 ng/mL), and sufficient 25(OH)D level is at least 50 nmol/L (20 ng/mL). 

To define correct answers regarding VD supplementation following VD dosage was used according to the guidelines: 

IOM, ESPGHAN, and Global Consensus Statement guidelines [20,27,37]: 400 international units (IU) for infants ages 12 months and younger, and 600 IU for older ones. 

US Endocrine Society guidelines [26]: 400–1000 IU for infants and 600–1000 IU for children aged 1–18 years. 

Central European Scientific Committee guidelines [25]: 400 IU/day for infants 0–6 months of age, 400–600 IU/day for infants 6–12 months of age and 600–1000 IU/day for older children between September and April or through the whole year if sufficient skin synthesis of VD is not ensured in the summer.

To define correct answers regarding hypovitaminosis D treatment following VD dosage was used according to the guidelines: 

Misra et al., guidelines [38]: 1000 IU/day of VD for children younger than 12 months and 5000 IU/day for children older than 12 months with a duration of therapy of 1 to 3 months.

US Endocrine Society guidelines [26]: For children aged 1–18 years 2000 IU/day for at least 6 weeks or with 50,000 IU once a week for at least 6 weeks to achieve a blood level of VD above 75 nmol/L, followed by supplemental therapy of 600–1000 IU/day. 

Central European Scientific Committee guidelines [25]: 1000–10,000 IU/day (~50,000 IU/week), depending on the patient’s age and body weight. Therapy duration usually varies from 1 to 3 months, depending on the severity of VD deficiency. 

Global Consensus Statement guidelines [37]: 2000 IU/day of VD for infants, and 3000–6000 IU/day for older children in the minimum period of 3 months. 

To further analyze our data, we evaluated differences in knowledge between different groups of pediatricians. Participants were divided into the following groups: Mediterranean (Albania, Bosnia and Herzegovina, Croatia, Cyprus, France, Greece, Italy, Montenegro, Spain, Turkey; and Portugal) vs. non-Mediterranean countries (all remaining countries); pediatricians who evaluate VD often and very often vs. those who do not; and different pediatric subspecializations. From this analysis, we excluded all participants who did not answer in one of the analyzed categories. 

Differences in knowledge between groups were analyzed using the χ^2^ test (*p*-value < 0.05 was considered statistically significant) via SPSS 24 (SPSS, Inc.; Chicago, IL, USA).

## 3. Results

### 3.1. Demographic Data and Professional Characteristics of Participants

During the study period, a total of 304 pediatricians from 33 European countries fulfilled the survey and were included in the study. There were no participants from two European countries (Finland and Slovakia). The participants were mostly female pediatricians (71.7%), and more than half of them (52.3%) had 11–30 years of practice. Almost 44% of them were general pediatricians, and among the subspecialists, endocrinologists and neonatologists were the most common ones. The largest number of participants worked in primary care (37%) or university hospitals (40.5%) (Table 1). 

Almost two-thirds of pediatricians (62.2%) at least sometimes evaluated 25(OH)D levels. Every fifth pediatrician (22.1%) very rarely evaluated 25(OH)D levels in their patients. ESPGHAN’s guidelines (42.8%) and the national guidelines (39.1%) were the most frequent ones used by our participants. However, some pediatricians were not using any guidelines (9.2%) (Table 1).

### 3.2. Definition of the Hypovitaminosis D and Indication for 25(OH)D Level Testing

Regarding the VD deficiency, 35.3% of the participants define it as 25(OH)D level <30 nmol/L (12 ng/mL), and 33.2% of them as 25(OH)D level <50 nmol/L (20 ng/mL). Both cut-off levels can be considered as correct according to the guidelines. Regarding VD insufficiency, pediatricians also chose two correct answers, 34.5% of them answered 30–50 nmol/L (12–20 ng/mL) and 30.3% of them answered 50–75 nmol/L (20–30 ng/mL) (Table 2). The most used indications for VD testing were as follows: osteomalacia/osteoporosis (62.2%), rickets (58.6%), history of fractures (56.3%), chronic kidney disease (53.3%), and autoimmune diseases (30.6%).

### 3.3. Vitamin D Supplementation Therapy

Almost all of the participants (98%) recommend supplementation with VD for infants. The highest number of participants used the same dose of VD supplementation during the entire year independent of the season of the year (40.4%).

Most of the participants knew the correct supplementation doses for infants, and among them, 61.2% recommended supplementation with 400 IU/day of VD for children younger than 12 months. In children older than 12 months their most recommended dose of VD for supplementation was 600–1000 IU/day (36.3%). However, a significant number of participants answered incorrectly, as they used VD dosages of 400 and 400–600 IU/day (22.5% and 18.4% of participants, respectively) for VD supplementation in children older than 12 months (Table 2). 

### 3.4. Dosage and Duration of the Therapy with VD in Patients with Verified Hypovitaminosis D

When asked about the 25(OH)D level cutoff point for the start of therapy with VD, 37.8% of pediatricians chose a 25(OH)D level of 30 nmol/L (12 ng/mL), an incorrect answer according to the guidelines, and 34.1% of them chose 50 nmol/L (20 ng/mL), which was a correct answer.

Participants mostly recommended daily low-dose VD supplements for the treatment of both VD insufficiency (51.3%) and deficiency (47%) to their patients. They also recommended more sunlight, calcium supplements, and dietary intakes of VD. 

The most commonly used therapeutic dose of VD by our participants for treating verified hypovitaminosis D in children older than 12 months was 1000 IU/day (37%), an incorrect answer according to the guidelines. A correct therapeutic VD dosage of 2000 IU/day was answered by 25.2% of the participants. 

For children younger than 12 months mostly used VD therapeutic dosage was <1000 IU/day (35%), which is incorrect. Two correct answers, 1000 and 2000 IU/day were chosen by 28.7% and 21.3% of the participants, respectively (Table 2). Usually, participants correctly treated patients with a therapeutic dose of VD for 3 months (34.5%), but there were 20% of them that give VD for 6 months (Table 2).

### 3.5. Difference between Groups in Knowledge about Hypovitaminosis D

There was no statistical difference between the frequency of correct answers regarding the definition of hypovitaminosis D in different subspecialization groups and in pediatricians who evaluate VD often and very often versus those who do not (Table 3). We found a significant difference between participants from the Mediterranean and non-Mediterranean countries concerning the frequency of correct answers regarding the diagnosis of VD deficiency (χ^2^ test, *p* = 0.046) (Table 3). 

There was no statistical difference between frequency of correct answers regarding supplementation with VD in different selected groups (Table 3).

There was no statistical difference between the frequency of correct answers regarding VD therapy in different subspecialization groups and in pediatricians who evaluate VD often and very often versus those who do not (Table 3). We found a significantly higher frequency of correct answers in the pediatricians from non-Mediterranean countries regarding the therapeutic doses of VD in children older than 12 months with hypovitaminosis D (χ^2^ test, *p* = 0.010). The same was found regarding the duration of VD therapy for treating verified hypovitaminosis, as well (χ^2^ test, *p* = 0.017) (Table 3).

## 4. Discussion

Hypovitaminosis D has recently been recognized as a worldwide pandemic, affecting the entire population [14,15,16]. This led to an increase in VD testing and supplementation [20,24,39,40]. Our research aimed to evaluate the knowledge and practice of pediatricians, who should be well educated regarding VD and hypovitaminosis D.

Our participants mainly evaluated 25(OH)D levels in patients with diseases related to the skeletal system. Those are diseases related to the classic VD actions that are well known for a longer period. The most common non-skeletal diseases that were indications for VD testing in our participants were autoimmune diseases. Until recently the aim of VD recommendations and guidelines was about its skeletal (classic) actions. Nowadays, experts expand the list of indications for testing 25(OH)D levels [25,26,41].

When asked about the correct 25(OH)D levels for the diagnosis of VD deficiency and insufficiency, most of our participants were divided between reference values in two selected answers. Reference values that were chosen by our participants coincide with the conflicting guidelines found in the literature. Guidelines found in the literature are also divided into two groups—on one side, Central European Scientific Committee on Vitamin D and United States Endocrine Society’s guidelines [25,26], and on the other, IOM’s [19,20], ESPGHAN’s [27], and the Consensus Statement on rickets management guidelines [37]. Our participant’s correct answers matched those in the mentioned guidelines. We can assert that the conflicting guidelines are probably the cause of the divergent pattern of knowledge and practice in our participants. These variations in the clinical diagnosis of hypovitaminosis D occur for various reasons, including conflicting professional recommendations and practice guidelines, unfamiliarity with those recommendations and guidelines, independent clinical judgment, or the tendency to default to laboratory-testing target levels [32].

Regarding supplemental VD therapy, our participants showed respectable knowledge regarding patients younger than 12 months. However, in older children, their knowledge around supplemental VD therapy declined. Guidelines have a very similar approach regarding the supplemental VD therapy. VD supplementation with 400 IU for infants aged 12 months and younger generally is the recommended dosage in the guidelines. For older children dosage usually varies between 600 and 1000 IU [20,25,26,27,37]. We can explain our respondent’s pattern with the fact that pediatricians, especially general pediatricians, have more experience with VD supplementation in infants. They usually prescribe supplemental VD to all infants as a standardized approach, but in older children they prescribe it less often (only in children with clinical indication or low sun exposure).

We also found conflicting knowledge regarding therapy with VD for patients with verified hypovitaminosis D. Primarily, only 34% of participants used the correct 25(OH)D level cutoff point for the start of VD therapy in verified hypovitaminosis D. Significant number of our participants (38%) used an incorrect 25(OH)D level cutoff point of 30 nmol/L that is not mentioned in the guidelines. That is probably due to the already mentioned conflicting reference values for the diagnosis of VD deficiency and insufficiency that are found in the guidelines. However, guidelines clearly state that the 25(OH)D level cutoff point for the therapy of hypovitaminosis D (deficiency and insufficiency included) is 50 nmol/L [25,38].

Only 39% of participants used the correct VD therapeutic dosage in children older than 12 months, and a large number of participants (37%) choose an incorrect VD dosage (1000 IU/day). Correct answers diverged, but the dominating correct answer was 2000 IU/day (25% of participants). Better knowledge was found regarding the children younger than 12 months where 50% of our participants used the correct VD therapeutic dosage. Correct answers were divided into two groups almost evenly (1000 and 2000 IU/day). However, the most common answer to this inquiry was the incorrect one, <1000 IU/day in 35% of participants which is worrying because this value is not mentioned in any of the guidelines. Regarding therapy duration, participants mostly answered correctly. However, there were still 20% of participants that administer VD therapy for 6 months, which is not consistent with the guidelines.

The guidelines and recommendations about therapy with VD are also conflicting. Mostly recommended dosage for infants is 1000 or 2000 IU/day of VD, and for older children between 3000 and 6000 IU/day, but some guidelines even recommend 10,000 IU/day (~50,000 IU/week) [25,26,37,38]. We consider that these discrepancies between guidelines and our participants’ knowledge are due to their lack of experience regarding the treatment of verified hypovitaminosis D. We also noticed a tendency to use low VD therapeutic dosages, lower than the ones mentioned in the guidelines. This is probably due to fear of VD intoxication and therefore participants are clinging to low doses of VD during longer periods. Additionally, there is a chance of confusing supplemental and therapeutic VD dosages, which can explain the tendency to lower VD dosage over longer periods.

When we compared knowledge between pediatricians from Mediterranean and non-Mediterranean countries, we found better knowledge from the latter ones. Pediatricians from non-Mediterranean countries probably have more experience in diagnosing and treating hypovitaminosis D because children from those countries have less annual sun exposure and the issue of hypovitaminosis D is more under the scope of their professional interest.

When we explored disagreements around the definition of VD deficiency/insufficiency and protocols for treatment and supplementation with VD between different pediatric sub-specialties, and groups of pediatricians with different frequency of evaluating VD, we did not find any statistically significant difference. We can surmise that frequent VD testing is not a valid precondition for satisfactory knowledge about VD and hypovitaminosis D. Poor knowledge regarding hypovitaminosis D can lead to unduly VD testing and unstandardized VD supplementation that is mentioned in the literature [20,24,39,40]. Surprisingly, non-existent knowledge difference between different pediatric sub-specialties was found. This can be due to divergent and small respondent sample.

## 5. Conclusions

Our participants showed the greatest knowledge regarding the definition of the hypovitaminosis D, vitamin D supplementation, and duration of therapy, but showed the least knowledge regarding the cut off-point for the start of the therapy and therapeutic doses used to treat hypovitaminosis D. Conflicting answers can be related to the differences between the guidelines. To resolve this problem uniform guidelines should be created, and those guidelines should be promoted by national or local pediatric associations. Additionally, further education about VD, hypovitaminosis D, and VD therapy standardization should be encouraged by European, national and local pediatric associations.

## 6. Strengths and Limitations of Our Study

The strength of our research is the very wide setting of our survey, involving pediatricians from 33 European countries, enabling a good representation of European pediatricians. Another strength is the research design that involves simple data collection, with an option to preserve participants’ anonymity. The survey questions are precise and cover the major concerns and differences in present clinical practice. However, the limitation of this study is the lack of a standardized, validated questionnaire, and for this reason we had to design our own survey. Another limitation is the self-selection of participants, considering only those motivated enough and interested in this topic responded to the survey. Our sample was not distributed evenly between European countries due to the variable participant response rate, and this can bias the findings. Additionally, we did not analyze the use of national guidelines. Therefore, further research in this field based on a larger and more evenly distributed sample between countries is warranted.

## Figures and Tables

**Table 1:** Study participants characteristics.

Characteristic	*n* (%)
**Gender, *N* = 304**	
Female	218 (71.7)
Male	86 (28.3)
**Country in which participant works, *N* = 304**	
Italy	51 (16.8)
Croatia	48 (15.8)
Slovenia	38 (12.5)
Serbia	27 (8.9)
Greece	22 (7.2)
Romania	11 (3.6)
Spain	9 (3)
Bulgaria	8 (2.6)
Albania	7 (2.3)
United Kingdom	7 (2.3)
Other	76 (25)
**Years of practice, *N* = 304**	
<5	43 (14.1)
6–10	37 (12.2)
10–30	159 (52.3)
>31	65 (21.4)
**Pediatric sub-specialization, *N* = 303**	
General pediatrician	133 (43.9)
Neonatology	31 (10.2)
Endocrinology	28 (9.2)
Gastroenterology	20 (6.6)
Intensive care	14 (4.6)
Nephrology	13 (4.3)
Allergology	12 (4)
Pulmonology	10 (3.3)
Other	42 (13.9)
**Workplace, *N* = 301**	
University hospital	122 (40.5)
Primary care	111 (36.9)
General hospital	50 (16.6)
Other institution	18 (6.0)
**Highest academic title, *N* = 301**	
MD	156 (51.8)
PhD	62 (20.6)
University professor	54 (18)
Master of Science	29 (9.6)
**How often do participants evaluate 25(OH)D levels in their patients, *N* = 281**	
Very rarely	62 (22.1)
Rarely	44 (15.7)
Sometimes	81 (28.8)
Often	70 (24.9)
Very often	24 (8.5)
Guidelines used, *N* = 454 *	
European Society for Pediatric Gastroenterology Hepatology and Nutrition guidelines	130 (42.8)
National guidelines	119 (39.1)
Global Consensus Recommendations on Prevention and Management of Nutritional Rickets	49 (16.1)
Local hospital guidelines	39 (12.8)
United States Endocrine Society’s guidelines	36 (11.8)
Central’s European Scientific Committee’s guidelines	31 (10.2)
Do not use guidelines	28 (9.2)
Institute of Medicine guidelines	14 (4.6)
Other	8 (2.6)

* Multiple answers type of question.

**Table 2:** Study participants knowledge indicated by percentage of correct answers * regarding diagnosis of hypovitaminosis D, vitamin D supplementation and therapy.

	*n* (%)
**Definition of the hypovitaminosis D**	
25(OH)D level for the diagnosis of VD deficiency, *N* = 238	
30 nmol/L (12 ng/mL)	84 (35.3)
50 nmol/L (20 ng/mL)	79 (33.2)
25(OH)D level for diagnosis of VD insufficiency, *N* = 238	
30–50 nmol/L (12–20 ng/mL)	82 (34.5)
50–75 nmol/L (20–30 ng/mL)	72 (30.3)
**Supplementation with vitamin D**	
VD doses for a supplementation in children younger than 12 months, *N* = 268	*n* (%)
400 IU/day	164 (61.2)
400–1000 IU/day	80 (29.9)
VD doses for a supplementation in children older than 12 months that are at risk ^†^ of developing hypovitaminosis D, *N* = 267	
600–1000 IU/day	97 (36.3)
600 IU/day	36 (13.5)
**Therapy with vitamin D**	
VD level cut off point to start with the therapeutic doses of VD therapy, N = 214	
50 nmol/L (20 ng/mL)	73 (34.1)
Therapeutic doses of VD used for treating verified hypovitaminosis D in children older than 12 months, *N* = 218	*n* (%)
2000 IU/day	55 (25.2)
5000 IU/day	20 (9.2)
6000 IU/day	8 (3.7)
50,000 IU/week	3 (1.4)
Therapeutic doses of VD used for treating verified hypovitaminosis D in children younger than 12 months, *N* = 216	
1000 IU/day	62 (28.7)
2000 IU/day	46 (21.3)
Duration of therapy with VD for treating verified hypovitaminosis D, *N* = 220	
3 months	76 (34.5)
2 months	33 (15)
1 month	16 (7.3)
1.5 months	12 (5.5)

* Answers that are according to the selected guidelines: Institute of Medicine (IOM) [20], Central European Scientific Committee [25], United States Endocrine Society [26], European Society for Pediatric Gastroenterology Hepatology and Nutrition (ESPGHAN) [27], Global Consensus Recommendations on Prevention and Management of Nutritional Rickets [37], Misra et al., guidelines [38]. ^†^ Children and adolescents with dark skin living in northern countries, children and adolescents without adequate sun exposure (excessive use of sunscreen with high SPF, staying indoors for much of the day, wearing clothes covering most of the skin, living in northern latitudes during wintertime), and obese children.

**Table 3:** Difference between groups in knowledge regarding hypovitaminosis.

Question	*n/N* (%)				*n/N* (%)			*n/N* (%)		
	Pediatric Sub-Specialization				Country of Working Place			Frequency of Evaluating VD		
	General Pediatrician	Endocrinology, Nephrology, Gastroenterology, Metabolic Diseases, Immunology	Else	*p* *	Mediterranean	Non-Mediterranean	*p* *	Evaluate VD Never, Rarely, and Sometimes	Evaluate VD Often and Very Often	*p* *
**Definition of the hypovitaminosis D**										
25(OH)D level for the diagnosis of VD deficiency	68/100 (68)	36/54 (66.7)	58/82 (70.7)	0.868	92/123 (74.8)	67/107 (62.6)	0.046	101/154 (65.6)	61/83 (73.5)	0.212
25(OH)D level for diagnosis of VD insufficiency	64/99 (64.6)	36/54 (66.7)	53/83 (63.9)	0.944	83/123 (67.5)	66/107 (61.7)	0.359	93/153 (60.8)	60/83 (72.3)	0.077
25(OH)D level cut off point to start with the therapeutic doses of VD therapy	29/87 (33.3)	14/48 (29.2)	30/77 (39)	0.513	41/113 (36.3)	27/93 (29)	0.271	47/134 (35.1)	25/79 (31.6)	0.609
**Supplementation with vitamin D**										
VD doses for a supplementation in children younger than 12 months	104/114 (91.2)	56/59 (94.9)	83/93 (89.2)	0.479	126/138 (91.3)	109/120 (90.8)	0.895	161/179 (89.9)	81/87 (93.1)	0.399
VD doses for a supplementation in children older than 12 months that are at risk of developing hypovitaminosis D	61/114 (53.5)	30/59 (50.8)	41/92 (44.6)	0.436	70/137 (51.1)	59/120 (49.2)	0.758	91/178 (51.1)	41/87 (47.1)	0.541
**Therapy with VD**										
Therapeutic doses of VD used for treating verified hypovitaminosis D in children older than 12 months	33/89 (37.1)	25/48 (52.1)	28/79 (35.4)	0.141	34/113 (30.1)	46/97 (47.4)	0.010	54/138 (39.1)	32/79 (40.5)	0.842
Therapeutic doses of VD used for treating verified hypovitaminosis D in children younger than 12 months	42/90 (46.7)	29/48 (60.4)	37/77 (48.1)	0.273	51/111 (45.9)	53/97 (54.6)	0.211	68/173 (49.6)	40/79 (50.6)	0.888
Duration of therapy with VD for treating verified hypovitaminosis	57/90 (63.3)	31/49 (63.3)	48/79 (60.8)	0.933	62/114 (54.4)	69/98 (70.4)	0.017	82/140 (58.6)	55/79 (69.6)	0.105

Correct answers *n/N* (%); * Chi squared.

## Data Availability

The data that support the findings of this study are available from the corresponding author, upon reasonable request.
